# Correlation between expression of epidermal growth factor receptor and adverse reactions after chemotherapy of advanced non-small-cell lung cancer

**DOI:** 10.12669/pjms.315.7939

**Published:** 2015

**Authors:** Zerui Hao, Chunyan Tian, Futang Yang, Jihong Zhang

**Affiliations:** 1 Zerui Hao, Department of Respiratory Diseases, The Second People’s Hospital of Liaocheng, Linqing 252601, China; 2 Chunyan Tian, Health Care & Gerontology Department, Yidu Central Hospital of Weifang, Qingzhou 262500, China; 3 Futang Yang, Department of Respiratory Diseases, The Second People’s Hospital of Liaocheng, Linqing 252601, China; 4 Jihong Zhang, Department of Critical Care Medicine (ICU), Weifang People’s Hospital, Weifang 261041, China

**Keywords:** Epidermal growth factor receptor, Advanced non-small-cell lung cancer, Chemotherapy, Adverse reaction, Correlation

## Abstract

**Objective:**

To analyze the correlation between expression of epidermal growth factor receptor (EGFR) and adverse reactions after chemotherapy of advanced non-small-cell lung cancer (NSCLC).

**Methods:**

A total of 120 NSCLC patients who were treated in our hospital from August 2009 to September 2011 were selected as an observation group, and another 120 healthy subjects were selected as a control group. EGFR expressions in both groups were detected. The observation group was subjected to combination chemotherapy, and their shorter- and long-term prognostic outcomes, adverse reactions and mortality were recorded. Meanwhile, correlation analysis was performed.

**Results:**

The observation group had significantly higher percentage and positive rate in EGFR expression than those of the control group (P<0.05). With increasing stage and lymphatic metastasis, the positive expression rate of EGFR rose significantly (P<0.05). In the observation group, the response rate of treatment was 62.5%, and the incidence rate of adverse reactions after chemotherapy was 28.3% (34/120). The 1-, 2- and 3-year survival rates were 38.3%, 15.0% and 10.0% respectively. Multiple Logistic regression analysis showed that TNM stage, lymphatic metastasis and positive EGFR expression were the main independent risk factors for post-chemotherapy adverse reactions (P<0.05).

**Conclusion:**

Advanced NSCLC was commonly accompanied by high EGFR expression. Although chemotherapy was able to improve the prognosis and survival rate, adverse reactions were also induced, being associated with the pathological characteristics and EGFR expressions of patients.

## INTRODUCTION

Lung cancer has become one of the most common malignant tumors worldwide, with the highest morbidity and mortality rates among males as well as the highest morality rate and the second highest morbidity rate among females.^1^,^2^ In most cases, lung cancer originates from bronchial mucous epithelia or glands, for which non-small-cell lung cancer (NSCLC) accounts over 80%.^3^,^4^ NSCLC is a worldwide health problem and a leading cause of cancer-related deaths. The prognosis of NSCLC is poor, which was mainly due to the later diagnosis of this cancer in almost 40% of the NSCLC patients.^5^ And the response rate to platinum-based regimen of advanced NSCLC was less than 30%, which is lower than that for ovarian cancer, esophageal cancer and head and neck cancer.^6^ When diagnosed, most patients with lung cancer are in the advanced stage due to insidious onset, lack of specific diagnostic protocol and susceptibility to invasion and metastasis, so the mortality rate is often high.^7^,^8^ Given the lack of surgical indications, patients with advanced lung cancer are mostly given chemotherapy. Although platinum-containing two-agent combination regimens can increase the survival rate of NSCLC patients, adverse reactions, including hematologic and non-hematologic toxicities, are inevitable. Meanwhile, adjuvant chemotherapy requires high tolerance.^9^

Epidermal growth factor receptor (EGFR) is a member of the ErbB family that mediates the signal transduction pathways of phosphatidylinositol-3 kinase, mitogen-activated protein kinase, together with signal transducer and activator of transcription 3 and 5. All the pathways are involved in cell proliferation, apoptosis escape and angiogenesis, with which malignant tumors are associated.^10^ Biologically speaking, EGFR is a glycoprotein receptor on the surface of cell membrane, with its expression determined by specific differentiation.^11^ It has been well-documented that EGFR is highly expressed on tumor cell surfaces. Generally, EGF first binds EGFR to form a dimer and phosphorylates the latter, thereby initiating the intracellular signal transduction system.^12^ Abnormally transduced in tumors mostly, EGFR has been paid particular attention in clinical examination and recent antitumor therapy.^13^,^14^ Therefore, it is necessary to reveal the differences between EGFR expression by observing post-chemotherapy adverse reactions. In this study, we explored the correlation between EGFR expression and adverse reactions after chemotherapy of advanced NSCLC.

## METHODS

### Subjects

This study was been approved by the ethics committee of Weifang People’s Hospital. Written consent was obtained from all subjects. A total of 120 NSCLC patients who were treated from August 2009 to September 2011 were selected as an observation group, and another 120 healthy subjects were selected as a control group.

### Section preparation and staining

The pathological specimens of the control and observation groups were collected, fixed in 10% formalin, embedded in paraffin, cut into 3 µm thick sections and stained. Antibody for EGFR: Rabbit anti-human EGFR monoclonal antibody (ZA-0505, Beijing Zhongshan Golden Bridge Biotechnology Co., Ltd.).

### Inclusion criteria

Pathologically diagnosed as NSCLC; with complete follow-up data; suitable for chemotherapy; 20-80 years old; prognostic survival time≥ 6 months; with normal hematological indices, liver and kidney functions; without receiving any chemotherapy or radiotherapy before diagnosis.

### Exclusion criteria

With undefined histological types; diagnosed as SCLC. The observation group comprised 68 males and 52 females, aged 43-78 years old (63.22±5.10 in average). The disease courses ranged from 3 months to 7 years, 3.22±1.89 in average.

### Histological typing

80 cases were of adenocarcinoma and 40 cases of squamous-cell carcinoma. TNM staging: 87 cases were of Stage III and 34 cases of Stage IV. Lymphatic metastasis: 80 cases had metastasis. The control group consisted of 66 males and 54 females, aged 44-79 years old (63.54±5.21 in average). The gender ratio and age of the two groups were similar (P>0.05).

### Treatment Methods

All NSCLC patients were administered platinum/vinorelbine. Vinorelbine tartrate injection was intravenously infused on the 1st and 8th days at the dose of 20-25 mg/m^2^, and cisplatin was intravenously infused on the 1st to 3rd days at the dose of 20-30 mg/m^2^. They were treated for 4 cycles, 21 days for each cycle.

### Observation Indices

Evaluation on therapeutic effects: Baseline tumor foci were subjected to regular imaging examination after treatment and the therapeutic effects were evaluated based on the changes of solid tumors as complete response (CR), partial response (PR), stable disease (SD) and progressive disease (PD). CR: All foci disappeared, without new ones. PR: The sum of major axes was shortened by ≥30%, or without new foci. SD: The sum of major axes was shortened by <30%, or without new foci. PD: The sum of major axes increased by ≥20%, or with one or multiple new foci. Response rate (RR), which was expressed as (CR + PR)/total case number, was used as the evaluation index.

### Adverse reactions

Adverse reactions, including gastrointestinal reactions, nervous system damage, allergic reactions, liver and kidney damages and blood diseases, were graded into 0-4.

The 1-, 2- and 3-year survival rates were observed.

### Detection of EGFR expression

Immunohistochemical analysis was performed for the pathological specimens collected before and after treatment by using rabbit anti-human EGFR monoclonal antibody (ZA-0505, Beijing Zhongshan Golden Bridge Biotechnology Co., Ltd.), immunohistochemical assay kit (SP-9001, Beijing Zhongshan Golden Bridge Biotechnology Co., Ltd.) and concentrated DAB kit (ZLI-9017, Sangon Biotech (Shanghai) Co., Ltd.). Labeled streptavidin-biotin technique was employed to conduct immunohistochemical staining. Primary antibody was replaced with PBS as the negative control, and known positive sample was used as the positive control. The proportion of positive cells was calculated according to the brownish yellow particles in the cytoplasm and on the cell membrane.

### Detection of EGFR expressions in peripheral blood

Fasting whole bloods (3 ml) of all patients were collected in the morning before chemotherapy, anticoagulated with heparin sodium, centrifuged at 3000 r/minutes for 10 minutes, from which the plasma was separated and subpackaged into 1.5 ml centrifuge tubes and stored at -80°C. For detection, the plasma sample (0.5 ml) was placed in a 1.5 ml centrifuge tube without anticoagulant, centrifuged at 12000 r/minutes for 10 minutes, from which the supernatant (300 µl) was collected and placed in a 10 ml centrifuge tube. Afterwards, 200 µl of 5% perchloric acid solution was added, and the mixture was shaken for 5 minutes, centrifuged at 12000 r/min for 20 min. The resulting supernatant (15 µl) was analyzed by using Agilent 1100 HPLC system (USA).

### Statistical Analysis

All data were analyzed by SPSS18.0. Stratified univariate analysis was performed by Chi-square test, and multivariate analysis was conducted by logistic regression. Inter-groups comparisons were carried out by using Chi-square test and t test. P<0.05 was considered statistically significant.

## RESULTS

### EGFR Expressions

**Fig.1:**
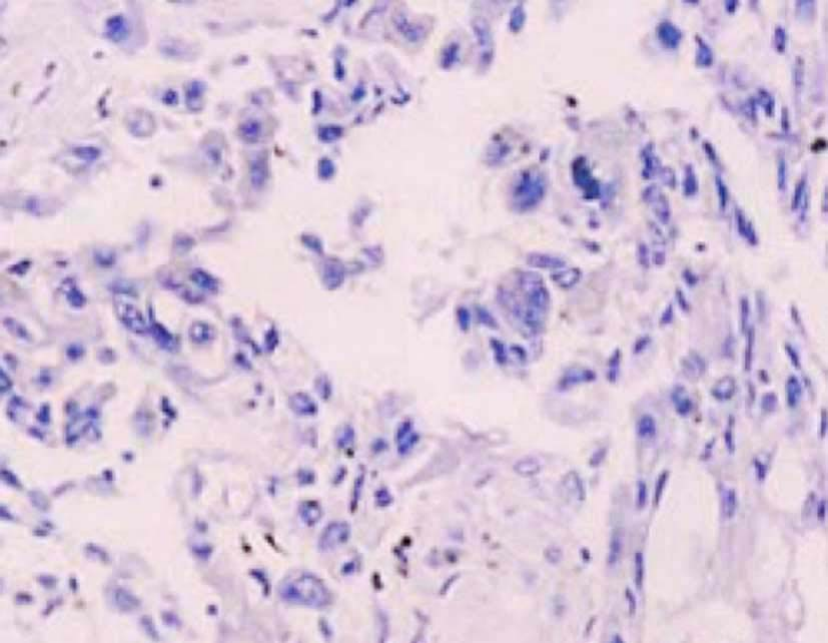
EGFR was negatively expressed in the control group (×100).

**Fig.2:**
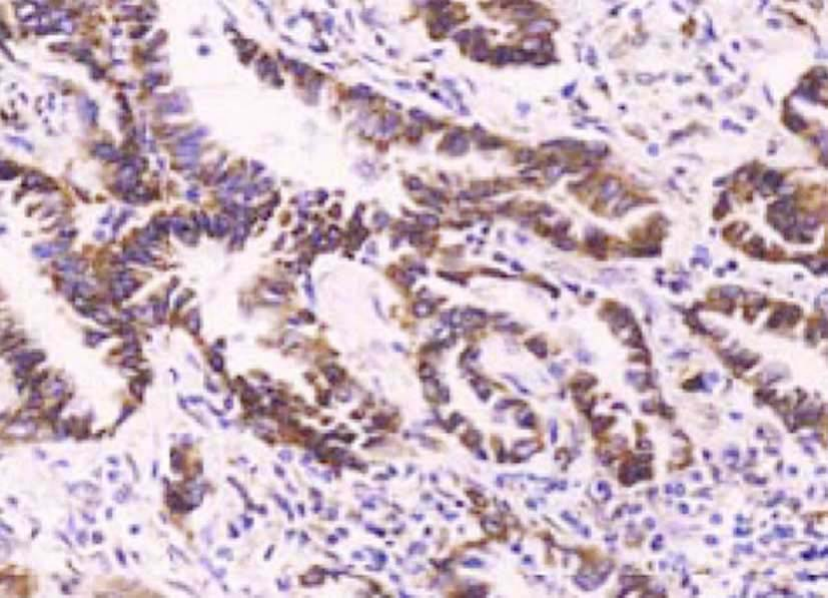
EGFR was positively expressed in the observation group (×100).

**Fig.3:**
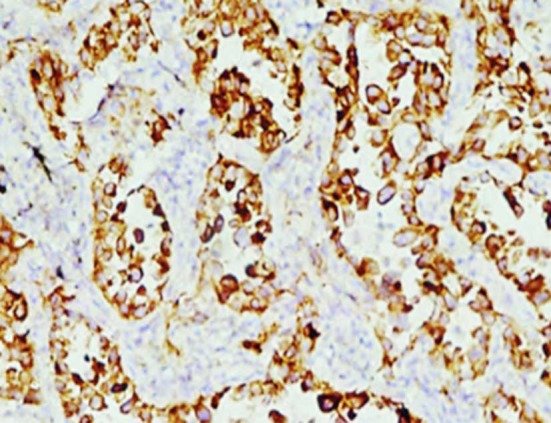
EGFR expression before chemotherapy (×100).

**Fig.4:**
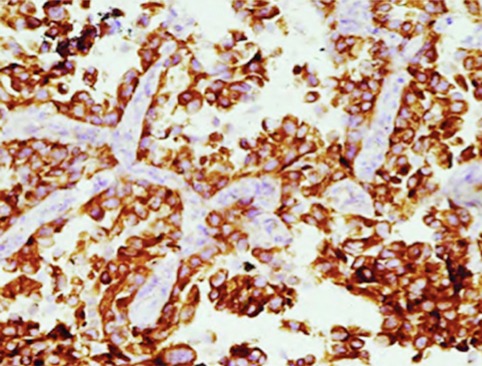
EGFR expression after chemotherapy (×100).

EGFR was positively expressed in the cytoplasm and on the cell membrane. The observation group had significantly higher percentage and positive rate in EGFR expression than those of the control group (P<0.05) (Table-I, Fig. 1 & 2). Besides, the expression of EGFR was up-regulated after chemotherapy treatment (Fig. 3 & 4).

**Table-I:** EGFR expressions.

Group	Case No. (n)	Percentage (%)	Positive rate (n)
Observation	120	58.45±25.44	76 (63.3%)
Control	120	4.55±1.22	3 (2.5%)
χ² or t		78.234	67.442
P		P<0.05	P<0.05

### Correlation between EGFR expression & pathological characteristics of advanced NSCLC

With increasing TNM stage and lymphatic metastasis, the positive expression rate of EGFR rose significantly (P<0.05) (Table-II).

**Table-II:** Correlation between EGFR expression & pathological characteristics of advanced NSCLC (n).

Pathological characteristic	Case No. (n=120)	Positive expression rate (n=76)	χ²	P
*Lymphatic metastasis*				
Yes	80	68 (85.0%)	16.442	<0.05
No	40	8 (20.0%)		
*TNM stage*				
III	87	48 (61.5%)	4.398	<0.05
IV	34	28 (82.3%)		

### Prognostic RR and adverse reactions

In the observation group, there were 35 CR cases, 40 PR cases, 30 SD cases and 15 PD cases, so RR was 62.5%. The incidence rate of adverse reactions after chemotherapy was 28.3% (34/120), including 12 cases of gastrointestinal reactions, 10 cases of nervous system damage, 8 cases of liver and kidney damages, 3 cases of allergic reactions and 1 case of blood disease (Table-III).

**Table-III:** Correlation between EGFR expressions in peripheral blood and post-chemotherapy adverse reactions (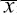
±s).

Adverse reaction	Case No.	EGFR (µg/L)	r value	P value
Gastrointestinal reactions	12		0.212	0.004
Grade I	6	1.323±0.644		
Grade II	5	2.094±0.323		
Grade III	1	2.435±0.114		
Nervous system damage	10		0.541	0.001
Grade I	7	1.453±0.432		
Grade II	2	2.321±0.773		
Grade III	1	2.785±0.195		
Liver and kidney damages	8		0.723	0.000
Grade I	6	2.003±0.174		
Grade II	2	2.121±0.913		
Grade III	0	2.792±0.934		
Allergic reactions	3		0.551	0.002
Grade I	2	2.003±0.174		
Grade II	1	2.121±0.913		
Grade III	0	2.792±0.934		
Blood disease	1		0.227	0.006
Grade I	1	3.123±0.432		
Grade II	0	3.725±0.674		
Grade III	0	4.241±0.872		

### Survival Rates

The 1-, 2- and 3-year survival rates of 120 patients were 38.5% (46/120), 15.0% (18/120) and 10.0% (12/120) respectively.

### Correlation between EGFR expression and post-chemotherapy adverse reactions

Univariate log-rank test showed that TNM stage, lymphatic metastasis, positive EGFR expression, age and smoking were possible risk factors for post-chemotherapy adverse reactions. Then multiple Logistic regression analysis showed that TNM stage, lymphatic metastasis and positive EGFR expression were the main independent risk factors (P<0.05) (Table-IV).

**Table-IV:** Correlation between EGFR expression and post-chemotherapy adverse reactions.

Variable	OR	95%CI	P
TNM stage	0.472	0.288-0.775	<0.05
Lymphatic metastasis	0.302	0.198-0.465	<0.05
Positive EGFR expression	1.782	1.187-2.543	<0.05

### Correlation between EGFR expression and basic physical conditions

The correlation between EGFR expression and basic physical conditions is summarized in Table-V.

**Table-V:** Correlation between EGFR expression and basic physical conditions (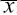
±s).

Item	Case No.	EGFR (µg/L)	P value
Gender			0.344
Male	68	4.372±0.673	
Female	52	3.595±0.367	
Age			0.557
<50	54	2.953±0.432	
>50	66	3.321±0.773	
TNM stage			0.041
I-II	35	1.614±0.219	
III-IV	85	5.787±0.135	
Lymphatic metastasis			0.037
No	40	2.113±0.191	
Yes	80	7.012±0.045	

## DISCUSSION

Lung cancer is the most dangerous malignant tumor that threatens human health and life and kills about 500 thousand people in China,^15^ among which NSCLC accounted for over 80% cases. Despite considerable research progress, 80% of lung cancer patients cannot be surgically treated upon diagnosis owing to insidious onset and lack of early diagnostic protocols. As a result, they have to receive chemotherapy, with unsatisfactory outcomes and low 3-year survival rates also.^16^ Mainly existing on the cell membrane, EGFR binds EGF and enters the cytoplasm through invagination and pinocytosis to transmit biological signals. Then EGFR is recycled onto the cell membrane after degradation of lysosomes. Generally, EGFR is highly expressed in tissues of gastric, esophageal and hepatic tumors, whereas it is barely expressed in corresponding normal tissues, suggesting that overexpression of EGFR may be related with tumor onset and progression.^17^

Compared with combination chemotherapy, multidisciplinary treatment for NSCLC can eliminate micrometastatic and residual foci, minimize the chance of recurrence, and improve long-term survival. Vinorelbine, as a cell-cycle specific plant-derived antitumor drug, is able to block tubulin polymerization and to induce its depolymerization, thus exerting remarkable killing effects on tumor cells.^18^ When combined with cisplatin, it improves prognosis by shrinking tumors, enhancing local blood supply and decreasing hypoxic cells. In this study, there were 35 CR cases, 40 PR cases, 30 SD cases and 15 PD cases in the observation group, so RR was 62.5%. The 1-, 2- and 3-year survival rates were 38.5% (46/120), 15.0% (18/120) and 10.0% (12/120) respectively.

Patients are bound to be affected by chemotherapy, mainly undergoing gastrointestinal, nervous system and allergic reactions. Commonly occurring immediately after several minutes of infusion, allergic reactions are manifested as rash in mild cases and chest distress, pain, shortness of breath in severe ones. The main symptoms of gastrointestinal reactions include hiccups, vomiting, diarrhea, inflammation of the upper digestive tract and intestinal ischemia. Skin reactions include local and systemic skin damages, mainly manifested as phlebitis, pain, erythema, as well as drug extravasation-induced skin shedding and necrosis. Moreover, hematopoietic system reactions mainly involve myelosuppression, and especially, neutropenia is closely associated with the dose and toxicity of chemotherapeutic agents.^19^

In this study, the incidence of adverse reactions after chemotherapy was 28.3% (34/120), including 12 cases of gastrointestinal reactions, 10 cases of nervous system damage, 8 cases of liver and kidney damages, 3 cases of allergic reactions and one case of blood disease. Accordingly, vinorelbine in combination with cisplatin mainly led to mild gastrointestinal reactions together with nervous system, liver and kidney damages which were alleviated thereafter.

Being a glycoprotein receptor, EGFR has the activity of tyrosine kinase, comprising 23 hydrophobic amino acid residues in the transmembrane region and 542 in the intracellular region. Binding between EGF and EGFR forms a dimer that is phosphorylated or transphosphorylated to produce biological signals, then activating several downstream signal transduction pathways and transducing them inside cell nuclei.^20^ It has previously been reported that EGFR is highly expressed in malignant tumors instead of in normal tissues.^21^ Similarly, the observation group herein had significantly higher percentage and positive rate in EGFR expression than those of the control group (P<0.05). In the meantime, the positive expression rate of EGFR rose significantly with increasing TNM stage and lymphatic metastasis (P<0.05). In general, tumors have higher growth and proliferative capacities and are more malignant with decreasing degree of differentiation and increasing TNM stage. EGFR can promote the metastasis of tumor cells partially by regulating integrin expression and functional subunit recombination, and it is invasion and metastasis that mainly result in the failure of lung cancer treatment and final death.^22^

Then multiple Logistic regression analysis herein showed that TNM stage, lymphatic metastasis and positive EGFR expression were the main independent risk factors (P<0.05). Since low degree of differentiation and absence of lymphatic metastasis benefit post-chemotherapy survival and reduce adverse reactions, TNM staging is indispensable before treatment.^23^ Furthermore; positive EGFR expression indirectly reflects the controlling of tumor pathological changes by drugs, the changes of which may predict the therapeutic effects of chemotherapy and post-treatment adverse reactions. The currently available EGFR-targeted drugs, including EGFR-tyrosine kinase inhibitors and monoclonal antibodies, can augment the activities of corresponding inhibitors and the susceptibility to gefitinib.^24^

In summary, advanced NSCLC was usually accompanied by high EGFR expression. Chemotherapy was capable of improving the prognosis and survival rate, but adverse reactions were also triggered, being related with the pathological characteristics and EGFR expressions of patients.
